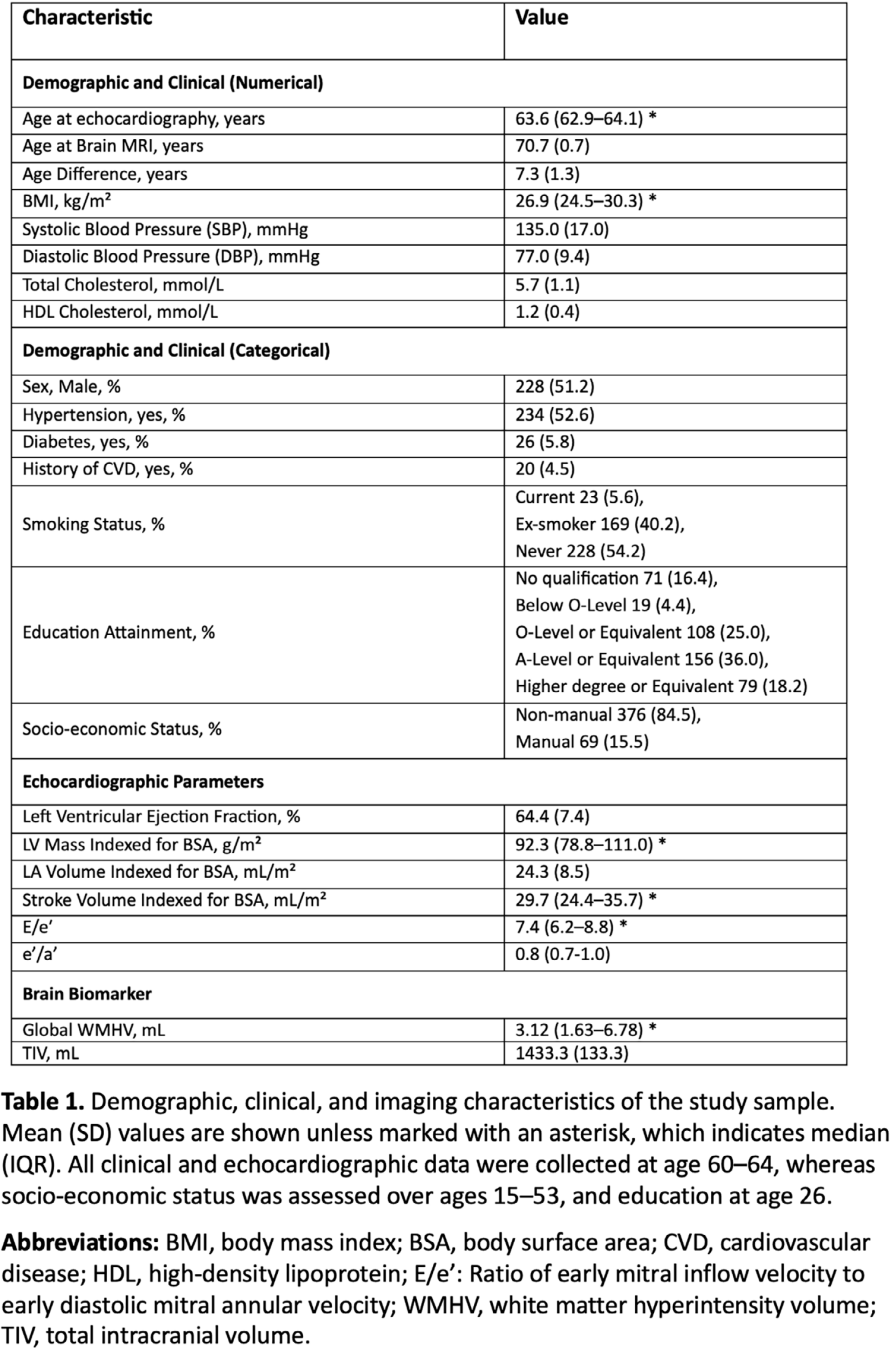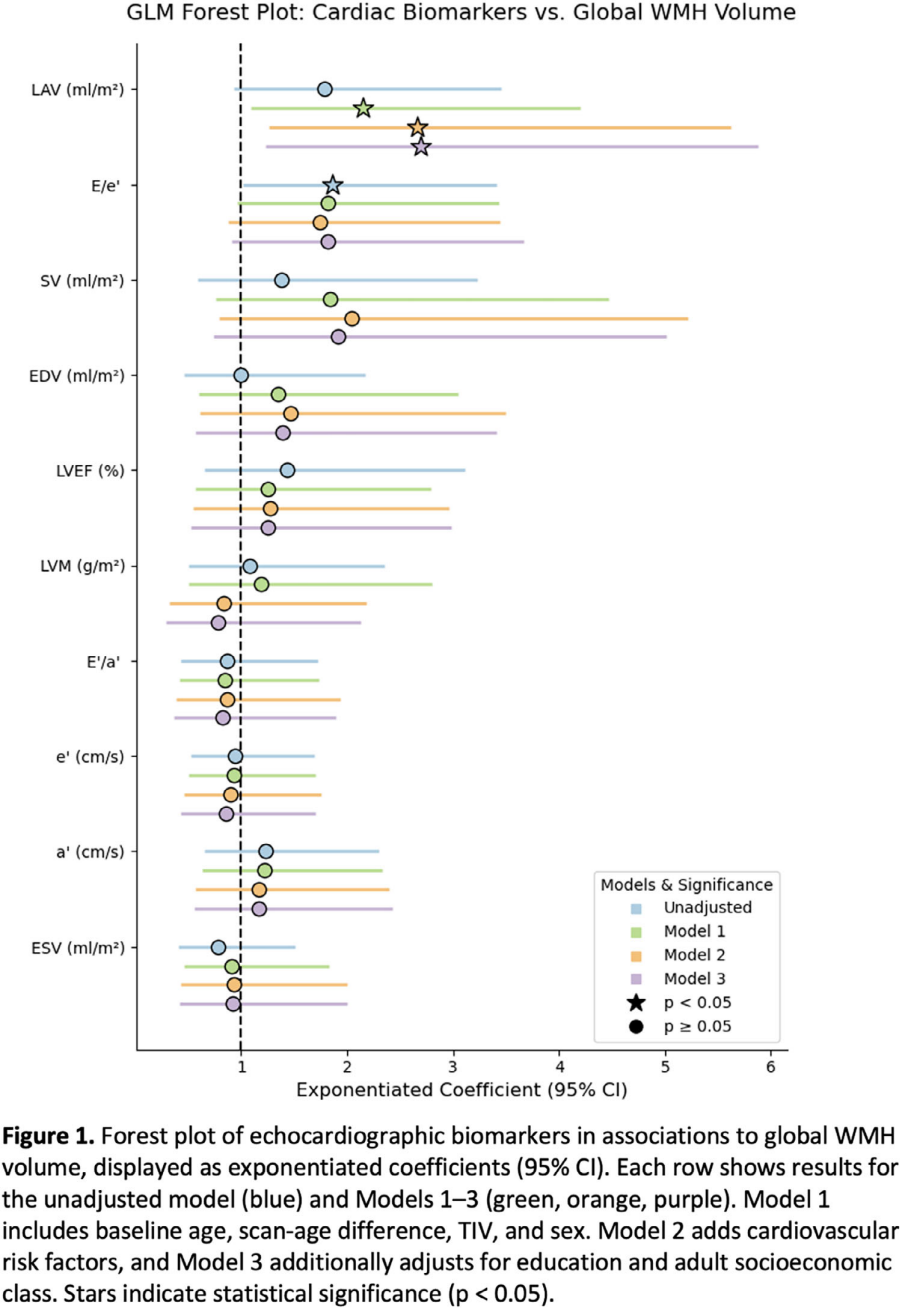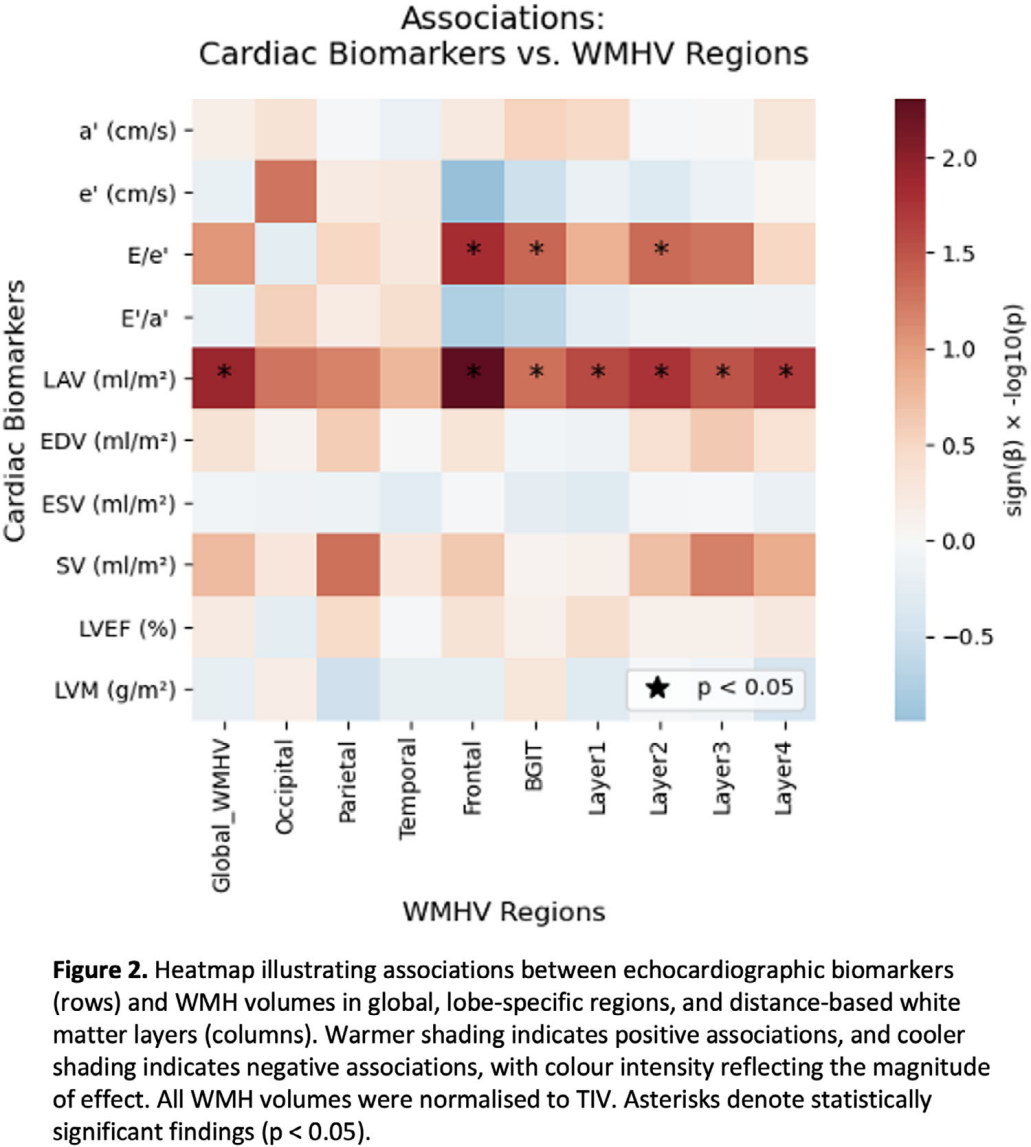# Associations of Echocardiographic Biomarkers with White Matter Hyperintensities: Insights from the British 1946 Birth Cohort

**DOI:** 10.1002/alz70856_100967

**Published:** 2025-12-25

**Authors:** Lucas He, Jo Barnes, Zerui Li, David M Cash, Alun D Hughes, Jonathan M Schott, Rhodri H Davies, Carole H Sudre

**Affiliations:** ^1^ MRC Unit for Lifelong Health and Ageing at UCL, London, United Kingdom; ^2^ Dementia Research Centre, UCL Queen Square Institute of Neurology, University College London, London, United Kingdom; ^3^ Department of Bioengineering, Imperial College London, London, United Kingdom; ^4^ MRC Unit for Lifelong Health and Ageing, Department of Population Science and Experimental Medicine, University College London, London, United Kingdom; ^5^ Institute of Cardiovascular Science, University College London, London, England, United Kingdom; ^6^ MRC Unit for Lifelong Health and Ageing, University College London, London, United Kingdom; ^7^ Centre for Medical Image Computing, Department of Medical Physics and Biomedical Engineering, University College London, London, United Kingdom

## Abstract

**Background:**

White matter hyperintensities (WMH) are established markers of presumed cerebrovascular disease and are associated with cognitive decline. Despite growing recognition of heart–brain interactions, longitudinal evidence linking cardiac function to WMH development remains limited, partly due to reliance on cross‐sectional designs. This study investigates whether midlife echocardiographic parameters were linked to subsequent WMH burden 5–7 years later in participants from the National Survey for Health and Development (NSHD) Neuroimaging sub‐study (Insight46).

**Method:**

We analysed data from 445 participants in the NSHD sub‐study. Echocardiographicmeasurements at age 60‐64 years included systolic, diastolic, and structural parameters. Brain MRI at age 69‐71 years enabled automated WMH (mm^3^) quantification globally and across regions/depths. Key demographical and clinical details are provided in Table 1. Missing data (7%) were imputed with the MICE method. Associations between echocardiographic parameter and WMH volumes were examined using generalised linear models with gamma distribution, adjusting for demographic, cardiovascular, and socioeconomic factors. We applied the Benjamini‐Hochberg procedure for multiple comparison correction. Potential non‐linear relationships were explored using quadratic models and generalised additive models.

**Result:**

Left atrial volume indexed for body surface area (LAV/BSA) showed the strongest association with global WMH (2.0% increase per unit, 95% CI 0.41–3.66%, *p* = 0.014), equating to approximately 60 mm^3^ increase. Figure 1 presents the forest plot summarising these associations across all echocardiographic biomarkers. Regional analyses indicated pronounced frontal lobe effects for both LAV/BSA (2.4% increase, *p* = 0.005) and E/eʹ ratio (7.9% increase, *p* = 0.017); LAV/BSA was associated with WMH across all white matter layers, whereas E/eʹ ratio was most evident in intermediate layers. Figure 2 provides a heat‐map illustrating these spatial patterns. End systolic volume showed a non‐linear association with WMH (quadratic term, *p* = 0.001), suggesting complex interactions

**Conclusion:**

In this study, echocardiographic parameters, particularly left atrial measures, demonstrated significant associations with subsequent WMH burden over 5–7 years. Although these findings remain observational and do not confirm causality, they support the integration of cardiac assessments into comprehensive risk profiling for age‐related brain changes. Future work should clarify whether interventions aiming to optimise cardiac health can mitigate WMH accumulation and related cognitive risks in later life.